# Parkinson’s Disease and Caregiving Roles, Demands, and Support Needs and Experiences: A Scoping Review

**DOI:** 10.3390/healthcare13010079

**Published:** 2025-01-04

**Authors:** Margaret L. Longacre, Lacey Roche, George C. Kueppers, Bart Buurman

**Affiliations:** 1 Department of Public Health, College of Health Sciences, Arcadia University, 241 Easton Hall, 450 S. Easton Rd., Glenside, PA 19038, USA; 2 National Alliance for Caregiving, 1730 Rhode Island Avenue NW, Suite 812, Washington, DC 20036, USA

**Keywords:** Parkinson’s disease, caregiving, racial and gender diversity, scoping review

## Abstract

**Background and Objectives:** A public health priority is the increasing number of persons with Parkinson’s disease (PwP), and the need to provide them with support. We sought to synthesize the experiences of relatives or friends—family caregivers—who provide such support. **Eligibility Criteria:** This study was a scoping literature review modeled by the PRISMA guidelines. The articles for this review fit the following inclusion criteria: (1) studies including the perspective of caregivers of PwP, (2) studies conducted in the United States, and (3) studies conducted between January 2019 to January 2024. **Sources of Evidence:** Articles were identified by searching the PubMed, EBSCO, and Ovid databases between January 2019 and January 2024. The search terms included the following: (Parkinson’s disease) AND (caregiver OR caregiving OR carer). **Results:** A total of 31 articles were included. Most of the included articles are descriptive (n = 26), including quantitative (n = 17), qualitative (n = 7), and mixed-methods studies (n = 2). Gender, race, and ethnicity were not consistently reported. Findings across studies demonstrated common roles of caregiving (e.g., assistance with personal care), extensive physical and mental health strains, social isolation, and work and financial strain. Benefit-finding was also evident among caregivers including a goal of securing the PwP dignity and comfort as the disease progressed. The studies of this review provide perspectives on benefits and challenges of caregiving in this context and caregiver resources. **Conclusions:** Future studies need to improve racial and gender-related diversity and address caregiver strain and health.

## 1. Introduction

Parkinson’s disease (PD) is the second most prevalent neurodegenerative disease in the United States (US) after Alzheimer’s disease—affecting over a million people—and the prevalence is only expected to increase by 2040 [1]. PD most notably features the loss of dopamine-secreting neurons in the substantia nigra in the basal ganglia, which results in symptoms related to deficits in motor function, including resting tremor, bradykinesia, gait disturbances, and rigidity [1]. While movement-related symptoms comprise many of the traits of PD, neuropsychiatric symptoms such as depression, anxiety, and psychosis are also seen in many cases of PD, especially in later stages of the disease [2]. Furthermore, there are no curative treatments for PD. Thus, there are many challenges for both the person(s) with Parkinson’s disease (PwP) and their caregiver(s)—supporting relatives and friends—as they manage symptoms.

Caregivers are essential contributors of care for PwP, and such care might be supplemented with external or paid support especially when a PwP’s needs advance [3]. It is well known that, in general, many caregivers experience caregiving-related emotional, physical, and financial strain [4]. Financial, emotional, and physical strain is also often interrelated with and perpetuates declines in psychological health (e.g., clinical anxiety and depression), especially when needs are not adequately met [5,6,7]. It is also suggested that by supporting caregivers—such as through resources and patient- and family-centered care—there might be better outcomes for the caregivers and their care recipients [8,9,10].

To adequately support caregivers, it is essential to understand experiences providing care in specific care contexts. Indeed, although caregiving tasks are consistent across care contexts, there are also variations in both roles and outcomes for caregivers [11,12]. Due to PD symptoms causing functional deficits in both motor and psychiatric domains, caregivers of PwP assume many and varied roles, including, but not limited to, emotional support, physical support (e.g., prevention of falls), and psychiatric care, as well as assisting with activities of daily living [13]. In-depth attention to the unique roles and related strain on caregivers in the PD context is pivotal to the future development of interventions, programs, or policies to improve the health and well-being outcomes of caregivers. Although the study of caregiving for PwP has increased, synthesis of the literature continues to be a need. Available literature reviews have primarily focused on caregiver burden [13,14] and have not been comprehensive in understanding the roles and other impacts that might contribute to health or burden outcomes. However, one comprehensive review was completed in 2014 [15], but, given advances in the field, an updated review is appropriate. Furthermore, previous literature reviews on caregivers have not always critically considered the representativeness of samples with respect to diversity in race, ethnicity, or gender identity. Such assessments provide important context of findings [16].

Thus, we conducted a scoping literature review to synthesize literature on the experience of caregiving for PwP broadly, including roles, personal strain, and health and psychosocial deficits. The primary research question is as follows: What are the primary roles and strains among caregivers for PwP, and how is their health impacted? Moreover, a secondary goal was to review the characteristics of samples among the studies to understand the scope of contribution to all caregivers for PwP. This secondary research question—i.e., How do studies differ with respect to the representativeness of socio-demographic characteristics?—was exploratory in nature.

## 2. Methods and Procedures

### 2.1. Study Design

This study is a scoping review that follows aspects of the Preferred Reporting Items for Systematic Reviews and Meta-Analyses (PRISMA) guidelines [17]. Elements of the review protocol are detailed in the methods or noted otherwise, including the research question (see Section 1), eligibility criteria, search strategy, data extraction process, and synthesis approach. This protocol was also registered with OSF Registries. Critical appraisal of individual sources of evidence included the synthesis of the overview of methodology and sample characteristics as summarized in Table 1, but no specific tool was used for critical appraisal. The review process consisted of screening identified articles for eligibility and synthesizing the data and information obtained from the eligible articles [17].

### 2.2. Eligibility Criteria

The articles for this review fit the following inclusion criteria: (1) studies involving the perspective of caregivers for PwP, (2) studies conducted in the US, and (3) studies conducted between January 2019 to January 2024. Exclusion criteria for articles were (1) studies that do not include the perspective of a caregiver for a PwP, (2) studies conducted outside of the US, (3) studies conducted before January 2019, and (4) studies that were systematic literature reviews, dissertations, or non-peer-reviewed materials. The purpose of only including studies conducted in the US was because of the potential impact that health and long-term care systems and policies have on caregiving. The five-year inclusion criteria also ensures current studies were obtained and analyzed for this review. Papers that had participants from other countries but had data from a distinct US population [46,47] were also included, but only included findings specific to the US population.

### 2.3. Procedures

The articles for this review were identified by searching PubMed, EBSCO, and Ovid databases. The search terms included: (Parkinson’s disease) AND (caregiver OR caregiving OR carer). The date range filter option was used with each database to include articles published between January 2019 and January 2024. Specifically for the EBSCO database, the Academic Search Ultimate database was searched using the “Full Text”, “Peer Reviewed”, “Academic Journal” (Publication Type), and “English” (Language) filter options in addition to the date range filter. The articles identified using PubMed, EBSCO, and Ovid databases were exported to EndNote in separate libraries. Using EndNote functionality, any duplicate articles obtained were removed.

The article list was initially screened by title, date range, and abstract. Articles seeming not to be relevant or that were published outside of the pre-identified date range were removed. Next, a secondary screening occurred by reading the abstract and full-text article to determine eligibility. Articles that did not fit the eligibility criteria based on further exploration were removed from the list. The article selection process was validated by a secondary reviewer to ensure that appropriate articles were included based on the eligibility criteria. For both the initial and secondary screening, an Excel table was used to track reasons for ineligibility, and this tracking tool was used by the reviewers to track and aid discussion if needed with respect to confirming eligibility.

### 2.4. Analysis Plan

Data from each eligible article were summarized into a data table (see Table 1). Findings across articles were also synthesized according to major themes as categorized in the results, including the following: (1) Perceived Caregiving Role and Responsibilities; (2) Health; (3) Work and Financial Impacts; and (4) Resources.

## 3. Results

**Figure 1:**
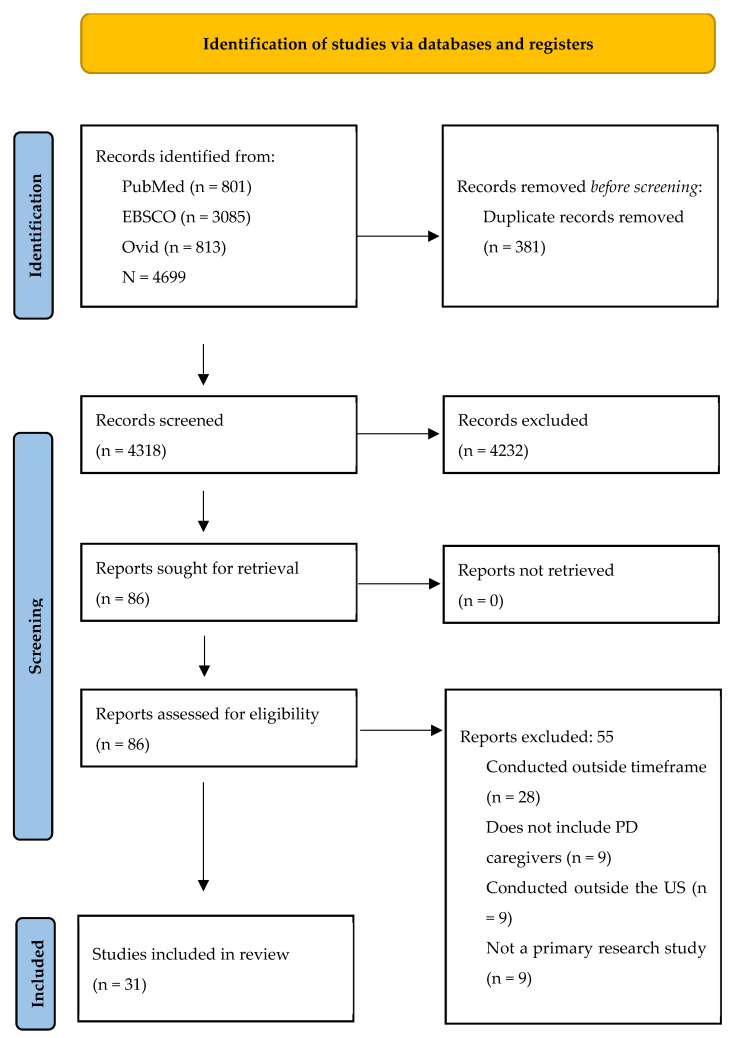
PRISMA Diagram of Search.

The initial search identified 4699 potential articles. After removing duplicates and conducting primary and secondary screenings, 31 articles were identified as eligible. Figure 1 outlines the PRISMA article identification process in detail. Most of the included articles are descriptive (n = 26), including quantitative (n = 17), qualitative (n = 7), or mixed-methods studies (n = 2). Of the quantitative studies, most were cross-sectional studies (n = 12) while a few were cohort studies (n = 5). The qualitative studies included semi-structured interviews (n = 6) and one case study. A few articles included studies with an intervention (n = 5), of which four were focused on reducing psychiatric symptoms of either the PwP, their caregiver, or both. Interventions focused on music therapy [36], mindfulness [27,39], mentorship [41], and education [30], with music therapy being found effective in reducing apathy in PwP and mindfulness effective in improving the reported relaxation of caregivers. Of the interventions that did not demonstrate significant differences among groups, satisfaction with the intervention was still high [30,41].

Table 1 provides information for each eligible study, including sample demographics, caregiving context, measurements, and outcomes. Of the 31 articles, age was the most common reported caregiver demographic or characteristic (n = 27), followed by gender (n = 24), race and/or ethnicity (n = 21), and relationship to the PwP (n = 18). Of the 24 articles that included gender, nearly all reported a large majority of female participants (average 76.1% female), with one study reporting the same number of male and female subjects (50% female, 50% male) [34], and a separate study reporting a slight male majority of 51.43% [42]. Of the 21 articles that reported race, all reported a majority of participants as white (average 93.1% white), with eight articles including 100% white participants [18,24,26,27,32,34,35,36]. Nuytemans et al. [26] was the only study to report a majority of participants as Hispanic/Latino for ethnicity. Lastly, of the articles that reported the relationship between the caregiver and the PwP, the most common reported relationship was a spouse or partner, followed by a child, then a sibling [19,20,21,24,25,26,30,31,33,35,36,38,39,40,44,45,46,47]. A spousal relationship was positively correlated with caregiver quality of life in one study [20].

### 3.1. Perceived Caregiving Role and Responsibilities

Multiple descriptive studies included in this review explored the perceived role and responsibilities of caregivers for PwP [19,21,25,28,35,38]. These roles include but are not limited to assistance with personal care, food preparation, obtaining and administering medications, mobility assistance, emotional support, transportation, home organization, handling of medical emergencies/crises, and financial responsibilities [21]. Through semi-structured interviews, Seshadri et al. [28] identified both challenges and benefits perceived by caregivers of PwP. The main challenges identified included the varied and unpredictable nature of symptom type and severity, requiring caregivers to be constantly alert and watchful [28]. Furthermore, the increase of symptomology and severity demonstrates the progression of the disease and inability to cure the disease to the caregiver, contributing to emotional challenges [28]. Other studies described perceived caregiving responsibilities in more detail, such as providing emotional and financial support [25], completing household tasks [35,38], and additional medical tasks, such as taking the PwP to medical appointments and assisting with medication adherence [38].

Although many challenges exist when caregiving for a PWP, caregivers also reported associated benefits. The caregivers in Seshadri et al. [28] reported that caregiving can allow the PwP to maintain as much dignity and comfort as possible and allows caregivers the opportunity for their own personal growth throughout the process. In another qualitative study interviewing caregivers after a dance class program with their care recipient, caregivers reported a similar theme of using their relationship with the care recipient to stay accountable and motivated in the dance class program [24].

### 3.2. Health

Several articles included in this review identified mental and physical health impacts of caregiving for PwP. Three studies specifically examined caregiver depression [23,29,47] and four studies specifically examined caregiver burden [22,29,42,45]. Overall, Smith et al. [45] found that the relationship between patient impairments and caregiver mental health was mediated by caregiver burden. The study conducted by Whiteley et al. [29] followed caregivers of people with PD over a two-year period and found that the percentage of caregivers with clinical levels of depression increased from 9.5% at baseline to 12.0% at the two-year follow up. Furthermore, it was found that caregiver burden was significantly greater after the two-year period as compared to baseline (*p* < 0.001), translating into 13 caregivers experiencing significant burden after two years. Finally, both subtypes of apathy demonstrated by PwP, low-interest and low-initiation, are noted to be correlated with burden and depression in caregivers [22,23].

Other studies examined the mental health impacts of caregiving more broadly, with participants reporting numerous symptoms associated with their caregiving role, including exhaustion [25], sadness/depression [20,25,38], anxiety/worry [18,25,38], grief [20], anger [38], and guilt [38]. Chahine et al. [21] found that feelings of resentment towards the care recipient were common emotional effects on caregivers and most predicted by symptoms related to disinhibition and agitation observed in the patient. Participants in Gallop et al. [38] noted that their relationships with the PwP and family were negatively impacted due to perceived lack of support. Respondents in Rigby et al. [20] reported that personality, behavior, and sleep symptoms caused the greatest emotional distress and decrease in quality of life (QOL) compared to any other symptoms due to caregivers’ relative inability to adequately prepare for these symptoms. Finally, mental health of caregivers can be negatively affected by virtue of social stigma, which was most experienced by caregivers with lower education level and/or social class, as well as those caring for PwP who had cognitive symptoms or symptoms related to bowel/bladder function [44].

Berger et al. [35] found that as PD progresses, the responsibilities associated with caregiving increase. As the caregiver role continues to expand, caregivers find it more difficult to stay socially engaged and participate in activities they enjoy. Seshadri et al. [19] found that the COVID-19 pandemic exacerbated this trend as it prompted the adoption of new caregiving responsibilities and external support was difficult or unsafe to obtain. Several studies focused specifically on social isolation as a specific contributor to caregiver burden [19,20,38]. In one study, social isolation was more likely to be observed in spousal caregivers compared to caregivers of adult children and caregivers not related to the patient [20]. Caregiving for PwP introduces significant social isolation to caregivers, resulting in the caregiver missing social occasions and being unable to connect with friends and family [38]. An exploration of social support systems in caregivers showed that strong family support predicted caregiver adaptability and response to crisis [46]. Social isolation can mediate many of the negative effects of caregiving, although decreased social support has been associated with increased feelings of self-efficacy which may have a protective effect against caregiver burden [20]. Seshadri et al. [19] also reported that the caregivers’ social isolation was especially evident during the COVID-19 pandemic, as the pressure to maintain isolation and subsequent inability to employ outside help made it difficult to balance self-care and caregiving duties. Chahine et al. [21] found that social effects on caregivers were most frequently predicted by symptoms of agitation and apathy and were more pronounced with younger caregivers.

Numerous physical health impacts from caregiving were also reported in studies, including difficulty sleeping [25,38], tiredness [38], inability to exercise [38], and physical pain [25]. Chahine et al. [21] reported that patient symptoms relating to nighttime behaviors, anxiety, depression, apathy, and agitation were most relevant in creating a physical strain on caregivers.

### 3.3. Work and Financial Impacts

Several studies reported employment status as a demographic characteristic of their study participants [21,27,34,36,38,40,43], including having a majority of participants indicating being retired [18,21,34,40,43], working full-time [38], or, as reported in one study, having an even distribution of participants working part-time and retired [27], and, lastly, simply reporting a majority of participants as “employed” [34].

Gallop et al. [38] examined the experience of caregiving on work and finances and reported that caregivers of PwP indicated the need to reduce the number of hours they work or quit their jobs to provide care. For caregivers working from home, additional time is spent overseeing the care recipient [38]. Caregivers also reported causes of financial challenges, including decreased net income because of working fewer hours and/or an increase in expenses, such as medications and other required medical devices (walkers, wheelchairs) [19,38]. Habermann et al. [3] briefly describes the financial burden caregivers face when obtaining external services that aid in keeping caregivers independent. These impacts were further exacerbated by the COVID-19 pandemic, with caregivers reporting the increased cost of outside care assistance and challenges managing their employment with caregiving responsibilities [19]. Compounding this financial challenge, Chahine et al. [21] found in a group of 450 caregivers that 98.7% of participants received no compensation for their caregiving services, despite its high demand on caregiver time and health.

### 3.4. Resources

Although limited, some studies of this review highlight the benefits and challenges of how resources were delivered to them. For example, Habermann et al. [3] reported on the positive impact that outside support services had for the caregivers, including helping families stay in their home, improving the safety of the care recipient, and promoting the independence of caregivers. However, resources (e.g., modifying one’s home) are often costly and can cause financial burden and strain [3]. One study uniquely explored an online tool to obtain resources [32]. When seeking out nutritional information with this tool, some caregivers reported that the tool was convenient and made it easy to obtain information. Others in the study reported challenges using the online modality, including an inability to apply the information found and a lack of access or knowledge in how to use the digital tool [32]. Other sources of information, specifically for psychosis-related symptoms, included the Internet, support groups, and workshops [31].

## 4. Discussion

PD symptoms require caregivers to assume a variety of roles to meet the daily living needs of care recipients and attempt to maintain their well-being. Given that caregivers for PwP assume a vital role in the well-being of their patients, interventions targeting the work of caregivers can potentially improve health outcomes. As demonstrated by Hazzan et al. [48], quality of life of caregivers has been correlated with improved outcomes—thus, understanding both met and unmet needs of caregivers of PwP offers a path towards superior care.

This scoping review provides a synthesis of studies exploring the experience of caregiving for PwP to assist in the goal of providing better care for PwP and their caregivers alike. Namely, the synthesis of the findings across studies included the identification of common roles, the existence of financial strain and health deficits, and the need for resources among caregivers for PwP. These three priority areas are highlighted below with respect to existing, relevant literature and to guide next steps. This review also sought to explore the representativeness of samples within studies, especially with respect to race and ethnicity, and findings suggest this is a vital area for improvement.

### 4.1. Caregiver Health

The findings provide insight on the health of caregivers for PwP, especially in the context of mental health. However, studies on the physical effects of caregiving for PwP are minimally explored. Although authors Gallop et al. [38] and Mantri et al. [25] report specific physical health impacts on caregivers, the severity of sleep loss, limit of exercise, and physical pain are not quantified. Despite physical tasks being a common component of care responsibilities, less than 10% of the articles included in the analysis bore any mention of the physical impacts of these activities. Uncovering more of the specifics of these physical symptoms can offer novel paths to support for caregivers. For example, elucidating typical injuries or activities that most frequently cause pain described in Mantri et al. [25] would be able to precisely guide interventions to alleviate pain for caregivers, whether it is orthotics or tools to alleviate the physical strain of certain repetitive motions.

The studies also pointed to an additional concern, which is isolation—and, though social in nature, might also result in physical decline. Findings regarding social isolation were complicated by the COVID-19 pandemic during the gathering of data. This is consistent with previous studies on caregivers of people with dementia or mild cognitive impairment, which showed that COVID restrictions exacerbated depression, anxiety, distress, caregiver burden, and quality of life [49]. However, the landscape of COVID restrictions in 2024 is far different from the restrictions of 2021 and 2022, which limits generalizability for these observations. However, this shows that caregivers, including PD caregivers, are an especially vulnerable population during public health crises.

Findings examining the mental health impacts associated with PD caregiving, specifically caregiver burden, align with similar patterns found in recent reviews. A review by Mosley et al. [14] examined caregiver burden for those caring for PwP and identified both depression and apathy as factors that increase caregiver burden. These factors of caregiver burden are further explored by the articles of this review, as apathy subtypes, including low-interest and low-initiation, were associated with caregiver burden [22,23]. As described by Mosley et al. [14], apathy includes a loss of emotionality and motivation, which are representative of these two subtypes. Both aspects of apathy exacerbate caregiver burden in different ways, as those demonstrating a decrease in emotionality reduce the positive feedback caregivers receive, while those demonstrating a decrease in motivation or low interest require additional prompting for tasks and activities, such as ADLs [14,50].

### 4.2. Caregiving-Related Work and Financial Strain

As identified in this scoping review, caregivers of PwP experience caregiving-related work and financial strain. Martinez-Martin et al. [51] found similar results by analyzing a large dataset consisting of data from 1998 to 2014. The analysis showed that caregivers of PwP had higher direct out-of-pocket costs, including both medical and prescription costs, and indirect costs as compared to the matched controls [51]. Additionally, the annual progressive income loss of caregivers of PwP was twice the loss of controls, indicating the lasting financial impact caregiving can cause [51]. A systematic review examining the financial impact of PD on PwP and their caregivers in the UK identified similar patterns, as caregivers reported having to reduce their working hours due to caregiving duties in addition to experiencing challenges when trying to obtain welfare services [52]. Furthermore, outside of PD, it is well documented over years that caregiving presents a financial strain and work impact as well as an emotional strain for many caregivers [4,53,54]. In 2021, Blue Cross Blue Shield reported the direct and indirect impact of caring as USD 264 billion and encompassing 51 million Americans [55]. This included work impacts such as disruption in hours or retiring early or quitting [4,55].

### 4.3. Resources Allocation

Only a few studies focused on resources for caregivers of PwP. Yet, a common need for caregivers of PwP is assistance in obtaining PD information and navigating the caregiver role as the disease progresses. Although physicians serve as a primary source of information for patients, physicians do not always ask caregivers about symptom management, such as psychosis [25] or aspiration status [37]. Caregivers are reported not to be comfortable mentioning such topics during appointments as to avoid embarrassment of the care recipient [25,31]. Caregivers might then seek PD information from other sources, including the Internet and support groups [31]. Aamodt et al. [13] identified in their review that this inability to obtain and utilize information from trusted sources contributes to caregiver stress and burden. Additionally, this challenge restricts caregivers’ ability to identify resources that may be available to them [13].

Identifying these gaps in information and resources creates opportunities for healthcare providers and organizations to include additional ways to provide information to caregivers, as in the study by LoBuono et al. [32], which evaluated the implementation of a digital platform for nutritional information. By furthering research in effective ways to meet the unique information needs of caregivers of PwP and anticipating the associated challenges and barriers caregivers may experience, providers can ensure information is provided efficiently and effectively.

### 4.4. Sample Characteristics for Studies

A secondary goal of this analysis was to explore the representativeness of samples among studies. The relative homogenous racial and ethnic compositions of the sample populations severely limits the generalizability of findings from these studies. All the studies in this literature review were majority white, with eight studies presenting with 100% white sample populations. While PD is more prevalent in white populations, Dahodwala et al. [56] reported in 2009 that PD occurs approximately in a 3:2:1 ratio in white, Hispanic, and Black populations. Moreover, Bailey and colleagues [57] concluded from their review of the PD patient literature that “minority populations should be emphasized in future research, as these patients are underrepresented in current research studies” (p. 839). Thus, further diversity in the PD caregiver population should be reasonably anticipated as well; and this should be a priority going forward for caregiving research. Furthermore, while this literature review was focused on the experience of US caregivers, a subset of studies included in this analysis include cultural comparisons of caregiver burden between Mexico and the US [44,45,46,47]. These studies show that cultural differences between the US and Mexico alone is an important factor in how caregiver burden is experienced. Therefore, future studies should better reflect the diversity of caregivers in the US to offer external validity to the findings.

## 5. Limitations

This scoping review allowed for the identification of needs and next steps with respect to PD caregiving, but there are several limitations to acknowledge. One limitation is the possibility of missing literature that might have fit the inclusion criteria because of the included search terms or databases. Although intentional, the exclusion of articles outside of the US limits the generalizability of the findings globally. We specifically intended to focus on US studies given the unique healthcare and long-term care systems or processes in the US that directly and indirectly influence caregiving support. Furthermore, socio-cultural factors are inherent within caregiving. There can be many interpersonal and socio-cultural beliefs and values that not only shape a care recipient’s experience but also that of the caregiver(s) (e.g., a caregiver’s choice in being a caregiver) [58,59]. Subsequent work can expand to conduct a comparison with non-US study findings. Lastly, multiple studies included in this literature review had small sample sizes and different forms of measurements, and, as noted in the results and discussion, had samples that lacked diversity especially with respect to race and ethnicity. These limitations could impact the generalizability of the findings with respect to the experiences and needs of caregivers. Despite these limitations, standardized processes were employed to avoid limitations and provide a valuable contribution to the literature to advance ongoing work and thought in this area of need for PwP and their caregivers.

## 6. Conclusions—Practical and Policy Implications

While the number studies of caregiver burden for PwP in the US is increasing in recent years, there remains gaps that must be explored. PD likely presents unique challenges not shared by other conditions that require caregiving, and an understanding of how these differences affect caregivers is essential to improving the long term care of PwP. Key areas of focus for research moving forward include improving recruitment strategies to include a more racially diverse study sample, examining the impact of PD symptom severity and progression on the physical health of caregivers, and identifying the information and resource needs of caregivers of PwP as well as the most effective ways to meet them.

Specifically, research should focus more broadly on the emotional, mental, social, and physical health of caregivers for PwP. A gap in focus exists with respect to the physical health of caregivers especially. Moreover, given findings of emotional and mental strain, especially during the COVID-19 pandemic, further exploration of social isolation and interconnection to mental and physical health is merited. It is also important that within the community and clinical context that caregivers are engaged and assessed for needs to support caregivers as well as their care recipients [60,61]. Second, caregivers for PwP are needing financial support because of the direct costs of PD (e.g., medical care costs) and indirect costs (e.g., work and retirement) loss. Such findings are similar in other care contexts and continue to highlight the need for expansive policies that allow for caregiving leave or choice in providing such care by having additional support. Adequate resources are needed to support improved health and well-being among caregivers for PwP and to reduce financial strain [4,62]. Given the sensitively of some topics of caregiving, these resources should be varied and include direct support as well as indirect learning opportunities (e.g., Internet-based support and education).

## Figures and Tables

**Table 1:** Eligible Studies and Characteristics.

First Author (Year)	Study Design	Purpose	Sample	Measurement	Outcomes
Phillips (2022) [18]	Cross-sectional study	Assess effects of stress on patient/caregiver dyads.	18 dyads of the Movement Disorder Clinic of Rehab Institute of the University of Louisville (n = 36) Marital status: 88.9% married heterosexual couples Race: 100% white Mean age: 63.67 for patients, 62.22 for caregivers Employment status: 16.7% full-time, 16.7% part-time, 33.3% retired	PD-specific stress—Impact of Event Scale Beck Anxiety Inventory, Beck Depression Inventory Cortisol—salivary cortisol at waking, 30 min after waking, bedtime Health status and QOL—Short-Form Health Survey	In both patients and caregivers, cortisol levels are elevated if their partner exhibits higher levels of PD-related distress.
Seshadri (2023) [19]	Semi-structured interviews	Explore challenges of the COVID-19 pandemic on strain and suggestions for support.	Caregivers belonging to Parkinson’s Foundation Centers of Excellence (n = 19) 95% spouse of patient, 5% adult child of patient Race: 89.5% white, 5.3% Black/African American, 5.3% unknown, 100% non-Hispanic/Latino	Qualitative Codes: -Changes in lifestyle during the pandemic -Daily challenges of caregiving during the pandemic -Social connections -Care partners’ health needs -Experiences with telehealth visits -Access to resources -Concerns and worries for the future	Caregivers reported: -Concern for patient contracting COVID at healthcare settings. -Not being able to attend appointments due to COVID restrictions. -Difficulty balancing personal employment demands with cost of care and potential risks of bringing in outside help. -Difficulty balancing caregiving and self-care due to increased difficulty of reaching outside support and new caregiving roles. -Desire for social connection while feeling pressure to maintain isolation.
Rigby (2021) [20]	Cross-sectional online survey	Compare caregiver experiences between Lewy Body Dementia (LBD), PD, and Alzheimer’s Disease (AD).	Caregivers on the email lists of various AD and PD groups (n = 515). Sample included DLB = 394, AD = 69, PDD = 61. Race: 96.2% white Gender: 90% women Relationship to patient: 80.6% spouse Education: 98.4% high school graduate	Marwit–Meuser Caregiver Grief Inventory Short Form Zarit Burden Interview PHQ-2 Depression Scale Quality of Life in Alzheimer’s Scale Medical Outcome Study Social Support Perceived Change Index Ryff Psychological Well-Being Scale Dementia Care Confidence scale Mastery and Self-Efficacy	PD caregivers were more likely to be spouses and to cohabitate with partner (*p* < 0.001, *p* = 0.03, respectively), which was correlated to higher caregiver QOL. QOL of caregiver/recipient, social support, well-being, mastery and care confidence more likely associated with higher caregiver education and inversely associated with disease severity. Social support higher in adult children caregivers compared to spouse caregivers. Personality, behavior, and sleep symptoms were the most affecting symptoms due to inability to adequately prepare for symptoms.
Chahine (2021) [21]	Cross-sectional online survey	Assess neuropsychiatric symptoms and demographic factors in caregiver burden.	Caregivers recruited from the Fox Insight study (n = 450) Mean age: 65.87 Gender: 74% female, 26% male Relation of caregiver: 84.9% spouse/partner, 10.2% parent, 1.8% sibling Mean duration of caregiving: 5.47 years Employed outside home: 31.1% Role of caregiver: 88% not paid, living with patient; 10.7% not paid, not living with patient; 0.9% paid and living with patient; 0.4% paid and not living with patient Caregiving responsibilities: 44.9% assist with personal care, 67.6% food preparations, 54.4% obtaining/administering prescribed medications, 44.9% mobility assistance, 92.4% emotional support, 68.9% transportation, 74.0% home organization, 68.7% handling crises/medical emergencies, 62.4% financial responsibilities	Caregiver Burden Inventory (CBI) Neuropsychiatric Inventory Questionnaire (NPI-Q)	Symptoms that contribute to domains of caregiver strain, listed from most to least relevant: Overall burden—agitation, anxiety, apathy, nighttime behaviors, hallucinations, and depression. Time burden—anxiety, hallucinations, female sex of caregiver, apathy, caregiving duration, nighttime behaviors. Life development burden—apathy, anxiety, nighttime behaviors, agitations, hallucinations. Physical burden—nighttime behaviors, anxiety, depression, apathy, agitation. Emotional burden—disinhibition, agitation, female sex of caregiver. Social burden—agitation, age, apathy.
Eglit (2021) [22]	Cohort study	Assess apathy subtypes (non-apathetic, low interest, low initiation) and effect on clinical characteristics of the disease and caregiver burden.	PwP from ongoing studies of PD at San Diego VA Health Care System at UCSD (n = 157) Race—93% white Age—67.6 Education—16.54 years (mean) Disease duration—66.1 months (mean)	Apathy Scale Caregiver Burden Scale	Both low-interest and low-initiation subtypes of apathy were associated with worse clinical presentation (cognitive and functional ability) and higher caregiver burden (*p* < 0.01).
Crowley (2022) [23]	Retrospective clinical cohort data review	Assess relationship between patient and caregiver ratings of neuropsychiatric symptoms in PD. Also determine if severity of depressive symptoms affects caregiver perceptions of other neuropsychiatric symptoms.	Patients with a confirmed PD diagnosis. Demographics only provided for patients (Mean age—64.3, Gender—74.2% male, Race—96.7% white, Education—Mean years = 14.2).	FrSBe severity of behaviors associated with frontal–subcortical dysfunction BDI-II	Positive association between depression and apathy (*p* = 0.006), disinhibition (*p* = 0.042) and difference between patient/caregiver rating of neuropsychiatric symptoms.
Prieto (2021) [24]	Semi-structured interviews with demographics survey following program	Obtain and describe the experiences of PwP and their care partners (CP) who participated in a Parkinson’s-focused community dance class.	5 PwP, 5 care partners (n = 10) Race: 100% white Age (PwP): 64 +/− 6.9 Gender (PwP): 3 female, 2 male Age (CP): 70 +/− 7.2 Gender (CP): 5 females CPs: 2 friends, 2 spouses, 1 mother	Participant characteristic questionnaire—demographic information Semi-structured interviews—focus on factors not observed during class and factors influencing class participation Observation of dance class (prior to interviews)	Three themes: -Keep Moving: CPs describe engaging in physical activity with the PwP to combat Parkinson’s symptoms and other comorbidities. -Compassion in Action: CPs motivated by their relationships with the PwP, spouse CP—means of enhancing relationship, friend CP—social responsibility. Class increased accountability between PwP and CP -Acceptance and Freedom in Dance: CPs expressed self-acceptance and support in the class setting; improved flexibility, balance, confidence in abilities, and independence. Active support group that allowed both social acceptance and self-evaluation.
Habermann (2020) [3]	Mixed methods study, semi-structured interviews and decision measures	Describe the types and timing of decisions made among dyads and to quantify and compare decision measures between PwP and caregivers.	15 dyads of patients and caregivers recruited from Parkinson’s support groups (n = 30) Mean age: 75.3 Race: 93.3% Caucasian Relation to patient: 100% spouse/partner Education level: mean years 15 years	Semi-structured interviews Decision measures: Decisional Self-Efficacy Scale Decisional Conflict Scale Decisional Regret Scale	Caregivers reported: -Outside services helped ensure the ability to stay in their homes, ensure patient safety, and ensure the maintenance of caregiver independence, although a financial burden was noted. -Moving into an assisted living community with a higher continuum of care was frequently discussed in tandem with other major decisions about living arrangements, such as moving closer to family for additional support. -Staying and maintaining living within the home was acknowledged as frequently being a product of a lack of knowledge about the Parkinson’s disease process. -Initiation of hospice was elected by two dyads and was discussed in the context of moving into assisted living, normally made in conjunction with adult children.
Mantri (2021) [25]	Cross-sectional survey	Describe the attitudes of care partners (CP) on Parkinson’s disease psychosis management and obtain their perspectives in developing materials for CPs on PwP.	145 CPs of PwP Age: 66.4 (avg) Gender: 75.9% female CP-PwP Relationship: 73.1% spouse, 17.9% children, 4.1% sibling	Survey—question about caregiving time constraints and role Neuropsychiatric Inventory Questionnaire (NPI-Q) -Additional questions on PDP (medications use, physician communication, sources of information on PDP) Caregiver Burden Index (CBI)	Responsibilities as caregiver: 91.7% emotional support, financial 79.3%, 79.3% reported spending time in caregiving role daily. Symptoms attributed to caregiving role: 73.1% reported exhaustion, 53.8% reported depression/sadness, 64.1% reported difficulty sleeping, 48.3% reported anxiety, 31% reported physical pain. 67.6% reported respite care, 54.5% reported exercising as favored coping strategies. 21.4% learned about PD from personal research, 15.9% from disease-specific organizations. 14.4% reported not wanting to discuss PD in front of PwP as a barrier in communication with physician; 17.2% did not want to embarrass the PwP, 18.6% reported there were more important symptoms to discuss. 68.3% of CPs reported wanting practical advance on managing PDP, 46.9% on a greater explanation of PDP, and 38.6% wanted options for treatment.
Nuytemans (2019) [26]	Cross-sectional survey	Identify willingness and motivations/barriers for PwP and caregivers (CGs) to participate in genomic research, with an exploration of differences between race and ethnicity.	162 participants (113 PwP, 46 CGs) CGs: 35 spouses/partners, 11 children, 3 other) Race/ethnicity (CGs): 29 white, Hispanic; 18 white, non-Hispanic Age (avg): 59.3 (white, Hispanic CGs), 59.9 (white, non-Hispanic CGs) Education: 58.6% (white, Hispanic CGs), 88.9% (white, non-Hispanic CGs)	Previously adapted (by authors) survey—assess influences on willingness to participate in genomic/genetic research; reasons for participating or not participating.	Approximately 91% of all participants noted they would participate in genomic study. 86.7% of white, non-Hispanic CGs selected “To help future generations with PD” as a reason for willingness to participate compared to 44% of white, Hispanic CGs. Other selected reasons: “To find new/better treatments for PD”—68% white, Hispanic CGs, 80% white, non-Hispanic CGs; “To improve science and knowledge about PD”—60% white, Hispanic CGs, 73.3% white, non-Hispanic CGs.
Seritan (2022) [27]	Non-randomized, cross-sectional intervention	Determine feasibility of Mindfulness-based cognitive therapy (MBCT) intervention in PwP and their CGs, differences in anxiety and depression for PwP, and changes in CG burden.	28 participants (24 PwP, 4 CGs) Age (CGs): 61.8 Gender (CGs): 25% male, 75% female Race: 100% white Employment status: 50% working part-time, 50% retired	MBCT intervention: 90 min sessions via Zoom, 8 weeks total Demographic survey Generalized Anxiety Disorder-7 item scale (GAD-7)—self-report instrument to measure anxiety and depressive symptoms over 2 weeks Patient Health Questionnaire-9 (PHQ-9)- self-report instrument to measure anxiety and depressive symptoms over 2 weeks Five-Facet Mindfulness Questionnaire-15 (FFMQ-15)—self-report measures of trait mindfulness Caregiver Self-Assessment Questionnaire (CSAQ)—self-report instrument that measures caregiver burden Satisfaction survey	75% of CGs attended at least 7 of 8 sessions. -Overall sample (PwP and CGs) showed significant reduction in depressive symptoms. -Overall sample (PwP and CGs) had significant GAD-7 and PHQ-9 score reductions and significant FFMQ-15 total score (*p* < 0.05). -96% of participants were satisfied or very satisfied with the course. 96% plan to continue to practice mindfulness.
Seshadri (2024) [28]	Semi-structured interviews	Understand PD care partners’ perceptions of their role and the challenges and rewards of caregiving.	16 care partners Age: 68.3 Gender: 31.25% male, 68.75% female Relationship to PWP: 87.5% spouse, 6.25% adult child, 6.25% sibling Race: 93.75% white, 6.25% Black Ethnicity: 100% non-Hispanic/Latino	Authors developed a semi-structured interview guide. Interviews conducted by tele-video, approx. 60 min.	Five Themes: -Unpredictability is the hardest part of caregiving—when disease symptoms would appear or the severity of symptoms; required being hypervigilant. -Disease progression and multiple symptoms contribute to care partners’ emotional challenges—symptomatology and disease progression left care partners feeling incompetent and helpless; realization of no cure or reversal of disease progression; watching decline of loved one with PD and changes in relationships/loss. -Caring for a family member is not a “burden”—care partners’ described caregiving duties as an “honor”; would rather use the works “stressful” and “difficult” than burdensome. -Caregiving is a partnership—joint decisions made between PwP and care partner, especially early in the disease; “care partnership” built upon communication and maintaining PwPs’ dignity. -Caregiving is an opportunity for personal satisfaction, joy, growth—care partners were intentional in findings moments of joy throughout disease progression; rewards included personal growth and contributed to the comfort of PwP.
Whiteley (2022) [29]	Prospective cohort study	Determine changes in care partner burden and depression over time and identify PwP and care partner characteristics that predict changes in burden and depression.	88 PwP and care partners Age (CPs): 63.3 Gender: 26 male, 62 female	Zarit Burden Inventory—care partner burden Geriatric Depression Scale Mattis Dementia Rating Scale Unified Parkinson’s Disease Rating Scale PwP—Hoehn and Yahr Disease Stage (H&Y)—motor exam (disease stage) All participants (PwP and CPs) were reassessed with same measure approx. 2 years after baseline	-27.6% of CPs met depression criteria at baseline, 27.3% at follow up. -ZBI score for CPs at baseline indicated mild–moderate burden (M = 13.12, SD = 12.35) with 20.5% CPs scoring clinically significant; follow up scores indicated mild–moderate burden (M = 17.60, SD = 15.39) with 33.0% CPs scoring clinically significant -13 CPs were not clinically burdened at baseline, but scoring so at follow up; 2 CPs were clinically burdened at baseline, but were no longer at follow up. -Mean depression scores were below clinically significant; 9.1% (baseline), 12.5% (follow up) scoring clinically significant -5 CPs’ depressive symptoms increased (reaching clinically significantly) at follow up -Significantly greater levels of CP burden (Z = −4.58, *p* < 0.001) and depression (Z = −2.41, *p* = 0.16) at follow up as compared to baseline -At follow up, 61.5% had increased burden (8% stable, 31% decreased) and depression increased for 45% (25% stable, 30% decreased) -Higher baseline H&Y (*p* = 0.013, 95% CI [0.06, 0.45]) and longer disease duration (*p* = 0.024, 95% CI [0.03, 0.43]) were significantly correlated with increased CP burden over 2 year follow up -Higher baseline disease stage (H&Y) best predicted increased CP burden (*p* = 0.027); higher baseline patient (PwP) depression best predicted increased CP depression (*p* = 0.002).
Schindler (2023) [30]	Prospective cohort study	Determine the TeleDREAMS intervention acceptability, benefits on health literacy and mood, and satisfaction with intervention for PwP and their care partner (CP); 8 week telehealth model to provide research education to promote research and advocacy in communities.	19 CPs Age: 66.7 (6.4) Gender: 68.4% female, 31.6% male Race: 21.1% Black, 10.5% Asian, 5.3% Hispanic or Latino, 63.2% white	Montreal Cognitive Assessment Tower of London (ToL) Rapid Estimate of Adult Literacy in Medicine (REALM) Beck Depression Inventory (BDI) Short-Form 12 (SF-12)—QoL Satisfaction	-CP Results: No changes noted in any performance measures post-intervention -Reported strong satisfaction with TeleDREAMS intervention; 10/11 CPs agreed or strongly agreed it enhanced their knowledge and skills about topics, would attend future events if offered, and found the content useful.
Mantri (2021) [31]	Semi-structured interviews	Understand the struggles and barriers for CPs of PwP (with disease psychosis) regarding provider communication, available community resources, and financial impacts of caregiving.	9 CPs Age: 61.4 (8.6) Gender: 100% female Relationship to PwP: 7 spouses, 1 sibling, 1 child Disease Duration: 0.5–18.5 years	Interview guide—areas of focus: duration and evolution of care needs, communication strategies with healthcare providers, coping mechanisms.	Characteristics of Psychosis: CPs identified psychosis triggers for PwP, including time of day and medications wearing off. Psychosis Impact on CPs: most CPs reported a large amount of time providing unpaid care (medication management, attending doctor appointments) and preventing them from other commitments. Some CPs recalled negative experiences: dropping out of school, suicidal ideation, losing temper, and denial. Coping Mechanisms: support groups, therapists, social workers, physicians; mindfulness meditation, solitary activities (reading). Navigation with Medical System: 7/10 CPs reported physicians did not routinely ask about psychosis. Alternative sources for psychosis information: support groups, workshops, television commercials, “Google”. CPs wanted to prevent PwP embarrassment by discussing it front of them.
LoBuono (2023) [32]	Semi-structured interviews	Determine facilitators and barriers in using digital health platforms for nutrition services for PwP and their CGs.	40 dyadic interviews (20 PwP, 20 CGs) Age: 66.4 +/− 13.0 Gender: 20% male, 80% female Race: 100% white Ethnicity: 100% non-Hispanic or Latino	24-item moderator guide—questions about assessing health information and use of digital health platforms Parkinson’s Disease Questionnaire (PDQ-39) Multidimensional Caregiver Strain Index European Union Wide Indicators of Digital Competence Dietary Screening Tool	Technology Benefits: Easy access to PD-related nutrition information, reduced burden and travel time, and helped maintain independence Other uses: sociability and entertainment Barriers: Information overload, privacy concerns, lack of access and/or knowledge of use, and frustration with modality
Henry (2020) [33]	Cross-sectional study	Assess the relationship between PwPs’ symptoms and CG QoL.	181 dyads Age: 64.04 (10.50) Gender: 77.9% female, 22.1% male Race/Ethnicity: 86.7% white, 7.7% Black, 2.2% Asian Relationship to PwP: 79% spouse, 14.4% child, 3.9% other family, 0.6% friend Frequency of CG visits: 86.7% daily, 3.9% 4–6 days/week, 6.1% 2–3 days/week CG hours/week: 113.90 (55.74)	Neuropsychological evaluation data Parkinson’s Disease Questionnaire for Caregivers (PDQ-Carer)—CG QoL Parkinson’s Disease Questionnaires (PDQ-39)—PwP QoL Non-Motor Symptoms Questionnaire—PwP QoL	All PD symptoms were positively correlated with each measure of CG QoL (all correlations *p* < 0.01). A correlation matrix identified (1) a larger relationship between female CG and CG strain and self-care and (2) a larger relationship between male PwP and CG QOL (i.e., strain, anxiety, depression).
Hicks (2019) [34]	Cross-sectional surveys	Assess dispositional mindfulness in relation to PD CG stress–health factors and assess for dyad dispositional mindfulness “crossover”.	18 dyads (18, PwP, 18 CGs) Age: 62.22 (10.435) Gender: 50% female, 50% male Race: 100% white Current employment status: 2 full-time, 1 part-time, 3 retired, 1 permanently disabled	Mindfulness Attention and Awareness Scale (MAAS) Perceived Stress Scale (PSS) Interpersonal Support Evaluation List (ISEL) Beck Depression Inventory (BDI) Pittsburgh Sleep Quality Index (PSQI) Health-Related Quality of Life (HRQOL)	Individuals with greater perceived stress reported less social support (*p* < 0.001). Individuals with less social support reported greater symptoms of depression (*p* < 0.001). Individuals with greater symptoms of depression reported poorer sleep quality (*p* < 0.01). Individuals with poorer sleep quality had poorer HRQOL (*p* < 0.01).
Berger (2019) [35]	Mixed-method prospective cohort study	To use social self-management to understand the experiences of a PD CP, benefits and challenges, and the CP’s ability to care for their partner and themself.	20 CPs Age: 67 (avg) Gender: 12 women, 8 men Race: 100% white Relationship to PwP: 100% spouses	Face-to-face interviews Quantitative and qualitative data via surveys—activity participants, social support, QoL	-Took an additional role as PwP’s abilities diminished (i.e., household tasks). Frustration in the time it took PwP to complete these tasks. -Difficult to engage in leisure activities and stay socially engaged with friends. -Strategies to support self and spouse included connecting with family and friends, planning and using formal and informal supports -Planning ahead was emphasized by 9 CPs. -Positive self-talk, keeping a positive attitude. -CPs provide physical assistance, verbal cues, and emotional support to spouses. Self-management and caregiving strategies: CPs reported using strategies that combined both self-management and caregiving strategies—CPs use strategies to help their PwP maintain their independence -CPs reported the difficulty in balancing their PwP’s independence and guilt for not assisting. Emotional impact: 11 CPs described the burden associated with caregiving, such as additional activities/tasks, and mixed emotions regarding taking leisure time for oneself and compassion for caring for their partner.
Shah-Zamora (2024) [36]	Prospective cohort study	Determine if music therapy can assist in decreasing apathy of PwP and improving caregiver burden. Music therapy intervention—developed in small groups, for one hour each week for a total of 12 weeks; PwP and CGs provided with instrument kits.	16 dyads Age: 62.6 +/− 11.1 Gender: 6.3% male, 93.8% female Race/Ethnicity: 100% white Relationship to PwP: 81.3% spouse, 12.5% child, 6.3% other Employment status: 50% employed, 31.3% retired, 18.8% unemployed or disabled	Apathy Scale (AS) Parkinson’s Disease Questionnaire (PDQ-8) Schwab and England Activities of Daily Living (SE-ADL) Beck Depression Inventory (BDI-II) Zarit Burden Interview (ZBI-12) Multidimensional Caregiver Strain Index Satisfaction Survey	Significant reduction in PwP apathy post-intervention (*p* = 0.002). No significant changes in CG burden or strain post-intervention (*p* = 0.142); when excluding the 2 CGs with low adherence to the intervention, a significant reduction in CG burden was identified (*p* = 0.031).
Brooks (2023) [37]	Case study	Share caregiver experience of an aspiration case by a PwP and reflection on aspects of care that could be improved.	74 y/o male with dementia, CP is spouse	n/a	PD patient died from aspiration PNA while hospitalized for slip and fall despite the caregiver providing an aspiration hospital safety kit provided by Parkinson’s Foundation. Based on CP’s feedback, the authors conclude: -Need for a standard screening for swallowing abilities -Standard protocol for medication management when dysphagia is present and minimizing PNA -Greater role for caregivers in the hospital setting
Gallop (2022) [38]	Cross-sectional study, semi-structured qualitative phone interviews	Explore the impact of caring for an individual with neurogenic orthostatic hypotension, a common comorbid condition seen in PD.	Caregivers recruited by specialist recruitment agency (n = 20) Age: 57.7 mean Gender: female 70% Relationship to PwP: partner 65%, child 30%, other 5% Employment status: full-time 40%, 20% part-time, retired 25%, 15% other	Coding domains: Caregiving duties Impact on physical health Emotional impact Impact on daily activities Impact on work Impact on social and leisure activities Impact on relationships Financial impact Impact on independence	Caregivers reported assisting with taking patient to appointments, cooking, assisting with medication. Expressed impaired sleep, tiredness, inability to exercise; anxiety, worry, depression, frustration, feeling bad for patient, anger, loneliness, guilt, feeling drained. Lack of time for self-care, household tasks, getting out of house, time to eat and drink. Quitting job to care for patient, working fewer hours, supervising patient while working from home. Unable to go out and have fun, missing social occasions, unable to see friends and family. Negative impact on relationship with PwP and family due to perceived lack of support. Reduced income due to lower hours of work obligation, high expenses due to medication, appts, walkers/wheelchairs. Need to supervise patient.
Shah-Zamora (2021) [39]	Mixed-method cohort study Intervention: 9 week mindfulness-based stress reduction course (9, 2.5 h classes); separate interventions for PwP and CPs	Determine the feasibility and impact of mindfulness-based stress reduction (MBSR) on PwP and CPs.	26 PwP, 11 CPs Age: 72 [67.5–74] Gender: 100% female Relationship to PwP: 100% spouses	Qualitative telephone survey—questions regarding stress management, most stressful PD symptom and impact on mood Parkinson’s Disease Quality of Life-39 Mindful Attention Awareness Scale (MAAS) Life Orientation Test-Revised (LOT-R)—assesses optimism for the future Non-motor Symptoms Scale (NMSS) Zarit Burden Inventory (ZBI)	Quantitative findings: -CPs did not demonstrate a significant decrease in ZBI after MBSR (*p* = 0.239) -CPs saw a significant increase in MAAS after MSBR (*p* = 0.022) -CPs saw a significant increase in LOT-R (*p* = 0.022) Qualitative findings: -Prior to intervention, no CPs reported using mindfulness for stress management; all 9/11 reported continued use of MBSR at 3-month follow up -All CPs reported improvements in their relationships (feeling calmer, less quick to anger)
Zimmerman (2022) [40]	Online survey	Compare PwPs’ experience of swallowing-related QoL and CGs perception of swallowing-related QoL.	36 dyads Age (CPs): 65.9 +/− 7.7 Gender: 75% female, 25% male Race: 91.7% white, 2.8% Black, 5.6% mixed/other Ethnicity: 0% Hispanic/Latino, 69.4% non- Hispanic/Latina, 5.6% other Relationship: 89% spouses Employment status: 30.6% employed, 2.8% homemaker, 0% unable to work, 66.7% retired	Swallowing Quality of Life Questionnaire (SWAL-QOL) Demographic and background clinical information—time since onset of disease, previous history of swallowing evaluations or treatment, CG burden, CG concern about PwPs’ cognition, CG level of care	Emotional burden was the most common challenge reported by CGs (12/36). Fatigue subsets of SWAL-QOL scores saw the biggest difference in PwP and CGs score (mean difference = 5.32). Food selection and mental health subsets of SWAL-QOL scores saw the smallest difference in PwP and CGs score (mean difference = 0.35 (food selection), 0.12 (mental health)).
Fleisher (2022) [41]	Non-randomized controlled trial	Assess intervention for homebound PD dyads and determine feasibility of intervention. Intervention was 16 weeks peer mentoring for homebound PwP and CGs (over a one year period); mentors (trained, experienced CGs) spoke with mentees for 30 min weekly by phone, videoconference, or in-person.	65 dyads Age (CGs): 64.1 (11.5) Gender: 78.5% female Race: 72.3% white, 15.4% Black, 9.2% Asian Ethnicity: 6.2% Hispanic/Latino	Multidimensional Caregiver Strain Index (MCSI)—physical, financial, interpersonal, demanding strain Share the Care—rates and frequency of mentor–mentee calls Client Satisfaction Inventory Short Form (CSI-SF) Hospital Anxiety and Depression Scale (HAADS) Caregiver Self-Efficacy Scale (CSES)	Intervention CGs’ strain was unchanged between baseline and one year (*p* = 0.51) while controls worsened slightly (*p* = 0.01). Mentees and mentors rated 100% of the visits as useful -Mean satisfaction—mentees = 90/100; mentors = 91/100. No significant change in caregiver strain in any study time point; percentage of CGs scoring in mild, moderate, severe strain categories did not shift over time.
Joyce (2022) [42]	Observational cohort study	Explore the relationship between caregiver burden and plasma suPAR levels in CGs of PwP.	CPs of PwP or other Parkinsonism conditions (n = 32) 21 identified as primary CGs, 11 identified as non-primary CGs Age: 63.2 (8.95) Gender: 51.43% male Race:91.43% white, 5.71% Asian, 2.86% other Ethnicity: 5.71% Latino/Hispanic, 94.29% non-Hispanic/Latino	Zarit Burden Inventory (ZBI)—CG burden CBC, CPR blood samples Cumulative Illness Rating Scale (CIRS) Hamilton Depression Rating Scale (HAM-D)	Primary CGs had significantly higher suPAR levels and ZBI-12 scores than non-primary CGs (*p* = 0.01; *p* < 0.0001).
Speelberg (2023) [43]	Cross-sectional, online survey	Explore the severity of burden of CPs of PwP during the COVID-19 pandemic.	Informal (unpaid) CPs (n = 273) Age: 63.8 (9.7) Gender: 72.9% female Race: 98.9% Caucasian, 0.7% American Indian/Alaska native, 1.1% Asian, 0.4% African American, 0.4% Native Pacific Islander Employment status: 21.6% full-time, 11.7% part-time, 61.2% retired, 4.8% unemployed	Modified Caregiver Strain Index (MCSI) Additional CP questions—changes due to COVID-19 pandemic, daily needs/services, social isolation, concern over health or health of PwP	Pandemic-related problems—factors contributing to CP burden: -61.2% reported social isolation -60.8% reported not being able to do the things that give joy -48.7% decline in health of PwP -40.3% concern over own health. MCSI: -Perceived difficult aspects selected most—36% reported a change in the PwP from their former self, 27% reported sleep disturbance -Selected least—7% financial strain, 12% work adjustments, 12% caregiving as inconvenient. Items that increased the most throughout the pandemic: -emotional strain (63%) -confinement (50%) -upsetting behavior of PwP (50%). Items that increased the least throughout the pandemic: -financial strain (13%) -family adjustments (34%) -sleep disturbances (36%).
Henry (2021) [44]	Retrospective cohort study, survey	Explore stigmas experienced by caregivers of PwP and the relationship to PD symptoms.	US caregivers only, recruited from PD clinics at public academic institutions (n = 105) Age: 68.7 Gender: 68.6% women, 31.4% men Race: 92.4% white, 2.9% Asian, 2.9% Black, multi-racial 1.0% Relationship to Patient: 93.3% spouse/significant other, 3.8% parent, 1.9% friend, 1.0% aunt/uncle Mean Hours of Care Per Week—59.38/week Mean Months as a Caregiver—46.78	MDS Unified Parkinson’s Disease Rating Scale—severity and characteristics of Parkinsonian symptomatology Affiliate Stigma Scale—caregiver internalization of stigma due to family member disability	Stigma associated with: -Education level (*β* = 0.27, *p* =.013) -Social class (*β* = −0.25, *p* = 0.021) -Mental health (*β* = 0.34, *p* = 0.010) -Bowel/bladder function (*β* = 0.33, *p* = 0.005)
Smith (2019) [45]	Cross-sectional study	Explore the relationship between motor/non-motor symptoms and caregiver burden, caregiver burden and mental health, and PD-related impairments and mental health.	US caregivers recruited from PMDC at Virginia Commonwealth University (n = 105) Mean age: 68.7 Caregiver gender: 68.6% female, 31.4% male Relation to patient: 93.3% spousal, 1.9% friend, 3.8% parent, 1.0% aunt/uncle Average months providing care: 46.8	Movement Disorder Society—Unified Parkinson’s Disease Rating Scale—PD-related impairments Zarit Burden Inventory PHQ-9 and GAD-7	Direct effect between motor impairments and mental health was b = −0.03 and was not significant (*p* = 0.097). Direct effect between non-motor impairments and mental health was b = −0.03. Caregiver burden mediates the association between non-motor impairments and caregiver mental health. Demonstrates that reducing caregiver burden is an important target for intervention.
Villaseñor (2020) [46]	Cross-sectional study	Examine the connections between family dynamics and coping.	US caregivers recruited from PMDC at Virginia Commonwealth University (n = 105) Mean age: 68.7 Hours of care a week: 59.38 Months as a caregiver:46.8 Gender: 68.6% female, 31.4% male Relation to patient: 93.3% spousal, 1.9% friend, 3.8% parent, 1.0% aunt/uncle	SCORE-15—family dynamic measurements across subscales of Strengths and Adaptability, Overwhelmed with Difficulties, Disrupted Communication Sense of Coherence scale—to measure caregiver sense of coherence, across subscales of Comprehensibility, Manageability, and meaning	Family strengths and adaptability predicted comprehensibility, manageability, and meaningfulness of CG. Unique predictors of manageability and meaningfulness included being overwhelmed by difficulties.
Perrin (2019) [47]	Cross-sectional study	Examine the connections between unmet needs and mental health of PD caregivers.	US caregivers recruited from PMDC at Virginia Commonwealth University (n = 105) Mean age: 68.7 Hours of care a week: 59.38 Months as a caregiver: 46.8 Gender: 68.6% female, 31.4% male Relation to patient: 93.3% spousal, 1.9% friend, 3.8% parent, 1.0% aunt/uncle	Family Needs Questionnaire (FNQ) GAD-7 PHQ-9	Unmet family needs explained 30.4% of variance in caregiver depression (*p* < 0.001), with emotional support (*p* = 0.004), instrumental support (*p* = 0.005) and community support network (*p* = 0.003) as unique predictors. Instrumental support included help keeping the house, getting a break from responsibilities.

## Data Availability

Not applicable.
